# Narratives of meaningful endurance – how migrant women escape the vicious cycle between health problems and unemployment

**DOI:** 10.1186/s40878-018-0088-0

**Published:** 2018-07-20

**Authors:** Jasmijn Slootjes, Saskia Keuzenkamp, Sawitri Saharso

**Affiliations:** 1 Berkeley Interdisciplinary Migration Initiative/D-Lab, UC Berkeley, Barrows Hall, Room 350H, Berkeley, CA 94720 USA; 2 Movisie - Netherlands Centre for Social Development, Catharijnesingel 47, 3511 GC Utrecht, The Netherlands; 3 Department of Sociology, Vrije Universiteit Amsterdam, Boelelaan 1105, 1081 HV Amsterdam, The Netherlands; 4 University of Humanistic Studies, Kromme Nieuwegracht 29, 3512 HD Utrecht, The Netherlands

**Keywords:** Sense of coherence, Narrative, Unemployment, Health, Migration, Coping, Women

## Abstract

Migrant women in Europe have a higher incidence of health problems and have disproportionately high unemployment rates. We examine how Dutch and Turkish, Moroccan and Surinamese first and second generation migrant women escape the vicious cycle between health problems and unemployment by using the theory of the Sense of Coherence (SOC). We study how SOC works and whether SOC is also applicable outside the domain of health. Our findings from life story interviews (*N* = 54) show that women can escape this vicious cycle through the meaningful reconstruction of adversity. Women can put a halt on the on-going negative chain reaction through focusing on the meaning and purpose of adversity. We name such life stories narratives of meaningful endurance, which are characterized by structure, authorship and meaningful reconstruction, in opposition to its counterpart, narratives of non-directional distress. The three respective components of SOC - comprehensibility, manageability and meaningfulness - enable the attainment of a narrative of meaningful endurance and individuals with a stronger SOC are more likely to tell narratives of meaningful endurance. Theoretical and policy implications of our findings are discussed.

*“All sorrows can be borne if we put them into a story”* (Arendt, 1958, p. 175).

## Introduction

Health and employment are mutually related throughout the life course (Paul & Moser, 2009; Schuring, Robroek, Lingsma, & Burdorf, 2015; Virtanen et al., 2005). Health problems have been found to have a negative effect on wages and hours worked (Pelkowski & Berger, 2004), and to reduce the likelihood to be employed altogether (Cai & Kalb, 2006; Chirikos, 1993; Pacheco, Page, & Webber, 2014). Yet, poor job characteristics (Griffin, Fuhrer, Stansfeld, & Marmot, 2002; Virtanen et al., 2005) and being unemployed (Dooley, Prause, & Ham-Rowbottom, 2000; Paul & Moser, 2009) can in turn have a negative effect on health. When considering the relation between health and employment, ethnic minority women form a particularly interesting case study. Women of Turkish, Moroccan and Surinamese origin living in the Netherlands have a higher incidence of different types of health problems (Gerritsen & Devillé, 2009; Grootveld et al., 2014; Ikram et al., 2015; Klaufus, Fassaert, & de Wit, 2014; Levecque & van Rossem, 2015) and have disproportionately high unemployment rates (Huijnk, Gijsberts, & Dagevos, 2014).^1^ With health problems leading to unemployment, and unemployment leading to more health problems, migrant women may be particularly at risk of becoming stuck in a vicious cycle or, in other words, a negative mutually reinforcing relation between health problems and unemployment.

Still, some individuals manage to escape this vicious cycle. The theory on the Sense of Coherence (SOC) provides a possible explanation for why some individuals are, and others are not, able to do so. SOC is a general orientation to life which represents the extent to which individuals (1) perceive arising issues as structured, predictable and explicable, (2) feel able to deal with arising issues, and (3) are willing and motivated to deal with these arising issues (Antonovsky, 1987). These three components are called comprehensibility, manageability and meaningfulness, which together determine whether an individual has a strong or a weak SOC. Antonovsky developed the theory of the Sense of Coherence in order to explain how individuals remain healthy despite the abundance of risk factors. This salutogenic orientation focuses on success factors rather than on risk factors. An elaborate review study of over 300 studies found that SOC is positively associated with various health related outcomes^2^ (Eriksson & Lindström, 2005).

Yet, the question *how* SOC operates and through which mechanisms has received much less attention in empirical research. According to Antonovsky, SOC partially operates through the coping process, arguing that individuals with a strong SOC are at an advantage at each stage of the coping process. They perceive arising issues as less threatening or benign, feel able and in possession of the resources to deal with arising issues and are motivated and willing to do so (Antonovsky, 1987). More recent studies suggest that narratives may play an important role in the coping process (Browne-Yung, Walker, & Luszcz, 2017; Carlick & Biley, 2004). In this study, as we will further explain below, we suggest that narrative is a mechanism through which SOC operates.

Despite the broad character of the SOC concept as a representation of an individual’s general orientation to life, it has so far only been used to explain well-being and different health-related outcomes (Eriksson & Lindström, 2005). However, it is likely that a strong SOC, with its important role in the coping process, is also applicable to other aspects of life, such as employment. In this study, we argue that SOC may play a role in the reinforcing relation between health problems and unemployment, thereby expanding the use of SOC outside the realm of health.

In this study, we set out to examine the mutually reinforcing relation between health problems^3^ and unemployment^4^ throughout the life course of women from various ethnic backgrounds living in the Netherlands. We aim to explain how women manage to escape the vicious cycle between health problems and unemployment by using the theory on the Sense of Coherence. We do so by conducting life story interviews with native Dutch women, and women of Turkish, Moroccan and Surinamese descent living in the Netherlands (*N* = 54). We first discuss the literature on the mechanisms through which SOC operates and how the literature on coping through narratives could offer a new perspective on how SOC operates. After discussing the background of the empirical research and elaborating how we use narrative analysis, we present the results in three main sections; We firstly go into the structural analysis of the narratives looking specifically at how the reinforcing relation between health and employment is narrated. Secondly, we examine how women escape these vicious cycles by discussing three types of narratives; narratives of meaningful endurance, narratives of non-directional distress and narratives in transition. Thirdly, we discuss the relation between these types of narratives and SOC. Lastly, we will discuss how our results may contribute to our understanding of and policies promoting migrant women to escape the vicious cycle between health problems and unemployment.

## Coping, narrative and the sense of coherence

Since Antonovsky wrote his seminal work ‘Unraveling the Mystery of Health’ and his suggestion that SOC works through influencing the coping process, the research on coping has expanded to studying how individuals cope through narratives. Narratives are usually understood as the story-like form through which people subjectively experience and give meaning to their daily lives and their actions. Previous research suggests that narratives enable individuals to recognize problems and to develop full understanding and meaning, which are essential parts of the coping process (Carlick & Biley, 2004). The interest in narration as a form of coping originates from research about (chronic) illness. These authors generally focus on how (chronic) illness threatens individuals’ identities and how narrating one’s life story can restore or transform individuals’ identities. Chronic illness is argued to create a *biographical disruption* which can be repaired through what researchers variably refer to as *biographical work to achieve biographical reinforcement* (Carricaburu & Pierret, 1995), *legitimation* (Bury, 1991), *knitting together ruptured identities (suturing)* (Riessman, 2015), or *narrative reconstruction* (Williams, 1984). Here, the focus is on the role of narrative in reconstructing order and continuity in individuals’ life stories which were interrupted and fragmented by the onset of chronic illness. Other authors expand the role of narrative in coping to its importance in the creation of meaning. As Bury (2001) notes; “Narratives feature prominently in the repair and restoring of meanings when they are threatened. Under conditions of adversity, individuals often feel a pressing need to re-examine and re-fashion their personal narratives in an attempt to maintain a sense of identity” (p. 264).

Frank identified three types of narratives in which individuals deal with biographical disruption through narrative. The *chaos story* reflects the type of narrative of individuals who are not able to knit together the split ends of illness disruption, resulting in a chaotic story about the perpetual nature of illness and pain. In contrast, *the restitution story* is a more linear narrative which focuses on how medical treatment restores health and how afterwards everything goes back to ‘normal’. Lastly, *the quest story* develops when a restoration of health and normalcy is impossible due to the nature of the illness. The quest story is about the reluctant acceptance of illness while focusing on the lessons that can be learned and how the self is transformed by illness (Frank, 1998).

Besides academic attention, narrative also found its way to the treatment room of psychologists where *narrative therapy* is now increasingly common (Etchison & Kleist, 2000; Gonçalves, Ribeiro, Silva, Mendes, & Sousa, 2016; Metcalf, 2017; Vetere & Dowling, 2016). Similar to the focus on narrative reconstruction in the coping literature, is narrative therapy’s focus on the creation of so-called *alternative stories* (Monk, John, Crocket, & Epston, 1997). These alternative stories are co-constructed to “replace disorganized or incoherent stories of self; lives become meaningful and coherent” (McAdams, 2006, p. 110).

In the more general literature on narrative analysis authors often speak about so-called *narrative coherence*. Narratives are more or less coherent depending on the extent to which they “(1) provide convincing causal explanations for the self, (2) reflect the richness of lived experience, and (3) advance socially-valued living action” (McAdams, 2006, p. 109). In an earlier study, McAdams (2001) distinguished between different types of coherence in narratives, addressing temporal, biographical, causal and thematic coherence. Creating coherence is often seen as the key function of narratives, yet some authors point out that the mission to find narrative coherence potentially marginalizes many types of narratives which are not characterized by coherence (Hyvärinen, Hydén, Saarenheimo, & Tamboukou, 2010). Instead of being a defining element of what is considered a narrative, it is a characteristic that may vary across narratives.

We hope this short excursion suffices to indicate the important parallels between the literature on coping through narratives, narrative therapy and narrative coherence on the one hand, and the theory of the Sense of Coherence on the other. Firstly, the focus on telling an intelligible story which is both structured and causally-ordered bears resemblance to the comprehensibility component of the Sense of Coherence. Individuals with strong comprehensibility perceive events as structured, ordered and causally linked. Secondly, the focus on the motives of characters, the creation of purpose and meaning parallel the meaningfulness component of the Sense of Coherence. Moreover, Antonovsky originally argued that the Sense of Coherence operates through the coping process. In the coping literature, narratives are now embraced as a form of coping with adversary life events like chronic illness. In conclusion, in this study we elaborate this line of thinking by examining whether narratives are a mechanism through which the Sense of Coherence operates and how narratives can help women to escape the vicious cycle between health problems and unemployment.

## Methods

### Participants and procedures

Life-story interviews (Atkinson, 1998, 2012) were held by the first author throughout 2015 with women of native Dutch (*N* = 8), Moroccan (*N* = 19), Turkish (*N* = 19) and Surinamese (*N* = 8) descent. We included women from Moroccan, Turkish and Surinamese descent as these women have a disproportionately high incidence of health problems (Gerritsen & Devillé, 2009) and relatively high unemployment rates (Huijnk et al., 2014), and could especially benefit from research focusing on how individuals may escape the vicious cycle between health problems and unemployment. In order to study the relation between health and employment, we only selected individuals who either suffered from regular headaches and/or regular neck, shoulder and back aches. We selected women with these health problems because they are the most common health complaints among ethnic minority and native Dutch women (Hessing-Wagner, 2006; van Lindert, Droomers, & Westert, 2004), are often caused by stress and may form an obstacle to employment.

We used purposive sampling to select respondents (*N* = 54) living in the four largest cities of the Netherlands (Amsterdam, Rotterdam, The Hague and Utrecht). The respondents were between 26 and 55 years old, with an average age of 39.5 years old. Besides the regular headaches and/or neck, shoulder and back aches, the extent of further psychological and physical health problems varied greatly. A fairly high percentage of 37% of the respondents indicated they are, or were, suffering from depression at some point during their life course. Lastly, 32% of the women in the sample were currently employed.

In the semi-structured life-story interviews women were asked to tell their life story and discuss their past, present and future and pay specific attention to the themes of health and employment. The interviewer aimed to interrupt as little as possible in order to not obstruct the natural narration of the life story, and only in order to ask for clarifications or get the interview back on track. Three women made use of an interpreter (at their request) due to their limited proficiency in Dutch. The interviews lasted from about 1 to 3 h, were audio-recorded, transcribed ad verbatim, made anonymous by using fictitious names and coded using Atlas.ti. After completing the interviews, we asked all participants to fill in the standardized and validated 13-item Sense of Coherence Questionnaire^5^ also known as the ‘Orientation to Life Questionnaire’ (Eriksson & Lindström, 2005). We used the validated translated Dutch version of the SOC-13 (Jellesma, Terwogt, & Rieffe, 2006). Questions were answered on a 7-point Likert scale and after reversing 6 items, possible scores varied between 13 and 91 with higher scores representing a stronger SOC (Cronbach’s α=.92).

### Analyses

The analyses consisted out of three steps. Firstly, we identify boundaries of different segments in the transcripts to analyse how segments function strategically in the narrative using structural analysis (Riessman, 1993). Secondly, we analyse the narratives specifically focusing on how women narrate the escape from or being stuck in a vicious cycle between health problems and unemployment. As the literature on SOC and coping through narratives emphasizes the importance of structure, causal order and meaning, we pay specific attention to these elements in the narratives. On the basis of the thematic analysis we are able to distinguish between two types of narratives that we label *narratives of meaningful endurance* (*N* = 32) and *narratives of non-directional distress* (*N* = 16)*,* which differ in structure, positioning of the narrator and meaningful reconstruction. This typology represents ideal types, an abstraction and simplification from reality, in which some narratives may incorporate elements of both ideal types but in which the majority more or less closely reflected one of these ideal types. Some life story interviews (*N* = 6) seemed to be positioned in between these two ideal types. We labelled them *narratives in transition*, reflecting the ongoing transformation of these narratives. In the third step of the analysis we compare the SOC scores between groups of individuals with different types of narratives using ANCOVA analysis in SPSS.

## Results

### The relation between health and employment - vicious and virtuous cycles

In the life-story interviews the women clearly narrate the relation between health and employment as mutually reinforcing. The structural analysis shows how women structure their life narratives in several chapters, either characterized by a positive or a negative reinforcing relation between health and employment. Inge, a 34-year old Dutch woman, provides an example of the positive reinforcing relation between health and employment; *“When the municipality gave me a house, everything became better, more stable. The headaches disappeared. And uhh, so I could focus on finding a job, and eventually succeeded. Now even my backache is gone. I always thought that I couldn’t work because of the pain in my back, but actually working helped solve my back problems haha!”.* We name this type of narrating of a positive reinforcing relationship between health, employment and other factors a ‘*virtuous cycle’*. Fatima, a 43-year old Moroccan marriage migrant, on the other hand, provides an example of a negative reinforcing relation: *“After the accident I had pain in my left arm, every day. The doctor could not help. I had to quit my job, I simply couldn’t do that cleaning work with that arm. And then, oh, everything got worse. I had no money, I worried. Also troubles sleeping, worrying, I even go to psychologist”.* We have named this negative reinforcing relation among health, employment and other factors a ‘*vicious cycle’.* A succession of reciprocal cause and effect in which several aspects of life intensify and aggravate each other, leading unavoidably to a deterioration of the situation. The life stories of the women we talked to contained both vicious and virtuous cycles at different stages in their lives.

Through grouping certain life events together and separating others, the women create structure and meaning in their narratives. The women demarcate the ending and beginning of these life chapters by clearly narrated turning points. These turning points provide an explanation for the shift of one life chapter to the next, either explaining a negative or a positive transition. Zeynep, a 49-year old Turkish marriage migrant, for example states: *“Yes, after the divorce it all started. [….] I was healthy, until… [claps in her hands] the divorce.”* Monique, a 43-year old Dutch woman, indicates it even more clearly describing it as a turning point; “*The day my mother died, that was.. That was the turning point. That’s when it all started, the headaches, not going to work and then losing my job, everything”.*

### Narrative typology

We did not only find differences in how women narrate the relation between health and employment, we also found distinct differences in how women narrate their entire life story depending on whether they currently perceive to be in a virtuous or vicious cycle. In general, women currently in a vicious cycle narrated their life story as what we term a ‘*narrative of non-directional distress*’ and women currently in a virtuous cycle narrated their life story as what we conceptualize as a ‘*narrative of meaningful endurance*’. These two types of narratives are so-called ideal-types. Some narratives were more difficult to categorize and highlighted how women are actively engaged in reconstructing and reshaping their life stories, which we name ‘*narratives in transition’*.

### Narratives of non-directional distress

The way women narrate being stuck in a vicious cycle have different aspects in common. We name this type of narrating *narratives of non-directional distress*. Zeynep’s story, a 49-year old Turkish marriage migrant, forms a good example; *“He [her ex-husband] went to other women. It was really hard.. I was depressed, going to a psychiatrist. Really not ok. Many problems. The children and me alone. I carry many things on my shoulders, huh? My parents are in Turkey, I am all alone here, my brother is also here, but.. You know, in that moment, then, uhh.. I just wanted to kill my ex-husband, really! My psychologist.. Uhh.. my head got a little weird. Yes in my brain, very busy brain. I went to the psychiatrist, he gave me medicine. I could’t sleep, I couldn’t eat, I lost 10 kilos. Yes, he went to other women.. ehh, I forgot the word. You know, I forget many things […] I don’t know. When I go home I cry, when I go to social services I cry, ehh, when walking I cry, when people ask me, ‘are you married?’, I cry. […] Nobody knows tomorrow, I don’t know. Insha’Allah, I hope I’ve had it all, I can’t take any more. […] what can I do? There is nothing I can do but await what will come.”* This excerpt is a good example of the unstructured and fragmented nature of a narrative of non-directional distress. Topics are switched, sentences not finished, and the temporal timeline switches. When asking her about her future, she indicates that no one knows what the future will bring, reflecting a lack of purpose or direction within her life story. Zeynep mentions how she carries a big load on her shoulders. This seems to reflect how she positions herself in her life narrative, as the bearer of a heavy load or the bearer of her story, not as the active author who influences the course of the story.

Another example is Saskia, a 35-year-old Dutch woman who developed a burnout and lost her job. *“The stress, the constant ‘I have to do this, I have to do that’.. Everything for my career. So there you are at 34 with a burnout. I couldn’t work anymore.. My friends.. I had always worked, all my friends were from work. I lost them too. I was alone, could hardly pay the mortgage. So I went to buy stuff, food, lots of Chinese food, for like 4 people. Debts, debts.. Just to think that those friendships were not real, they dropped me like a hot brick when I lost my job. None of them call me. People only care if you have money, if you are successful. They ran as soon as they knew. Well, it was all a lie. All that stress and hard work for nothing, I don’t buy nothing for it now.”* Interesting in this example is how previous experiences which used to feel meaningful, lost their meaning and purpose. All her hard work resulted in developing a burnout, losing her job and friends. *“I always did what I was supposed to do, get good grades, get a good job, work hard.. It got me nowhere. Now I don’t know who to listen to anymore.”* In this last excerpt Saskia positions herself as a passive actor in her own life story, doing what she is supposed to, not as the author of her own life story.

The previous excerpts are exemplary for women with a narrative of non-directional distress, which all seem to share some important characteristics. Firstly, these narratives were fragmented and unstructured, obstructing the causal links between different events and experiences. Secondly, the narrator tends to position herself as a passive character who has limited authorship over her life story, which is presented as determined by mostly external factors. Lastly, distress and hardship are described without attaching any meaning or purpose to it. These life stories thus come to resemble mere enumerations of difficulties and suffering, which lack purpose or direction.

### Narratives of meaningful endurance

How women narrate the escape from vicious cycles can be characterized by the structured nature of the narrative, the clear focus on authorship of the narrator and the meaningful reconstruction of hardship. We label this type of stories *narratives of meaningful endurance*. A clear example of such a narrative is the story of Assila, who is a 36-year-old Moroccan marriage migrant. Her husband is addicted to gambling and alcohol and is sometimes violent. “*I was told a beautiful future was waiting for me in the Netherlands, but no, nothing like that. No, he was a bad man, drinking, drinking, gambling, never money. He would hit me and the children, we were always scared. It was bad, depression, not sleeping, always pain in my head, always, exactly here […] I told my brothers, my father. But they said no, no divorce. I knew they would never talk to me again, never see me. […] Divorce meant losing everyone, family, the house, everything. […] The divorce was very painful. It was hard to be alone. But I did this for my children. I didn’t want them to be afraid anymore. For them I can do anything, nothing is hard, when it’s for them. It is worth it […] Now if I look back, I am proud. I am strong. I can take care of myself, of us. It is still hard, every day. But I know why I did it.”* Assila reconstructs her struggles as being suffered through for a purpose, her children, thereby narrating her hardship as being meaningful. In doing so, she is able to stop the chain reaction of adversity. If Assila could not attach a meaningful purpose to the painful and hard experience of the divorce, her pain and effort would be meaningless. However, she endured because she wanted to protect her children. By focusing on this meaningful purpose, Assila manages to halt the chain reaction of adversity by narrating her experiences in a narrative of meaningful endurance.

Another example is Anna, a 36-year-old Dutch-Surinamese woman, who is currently employed, but has suffered through multiple depressions and two burn-outs in the past partly due to identity struggles. *“I remember when I was 12 and visiting Suriname. There was a woman in the supermarket and she called me a bounty. Like, black on the outside but white on the inside […] After that I really started struggling with that issue, trying to find out who I am and where I belong. […] And yeah, then all these depressions and burn-outs started […] I especially want to contribute to the multicultural society and help people with a bicultural background. Then I think yes, I can help achieve that by using my background and what I’ve been through. […] That is really important to me, that is really my goal […] My biggest problem turned out to be my biggest source of inspiration. Haha. Yes, the circle is complete now, huh?”* Anna reconstructs her struggles and depressions as being suffered through for a purpose, thereby reconstructing her hardship as being meaningful. Her struggles with her identity inspired her major goal in life. It did not only inspire her and provide her with direction in life, Anna indicates that her background and her struggles formed a learning experience, thereby focusing on the ‘silver lining’ of the hardship she endured. Through the reconstruction from unordered chaos and suffering into a learning experience and a source of inspiration which guide her to a meaningful future, these events become less negative and enable her to escape from the vicious cycle of health problems and unemployment.

If Anna or Assila were interviewed several years ago, their narratives would be qualified as a narrative of non-directional distress. However, they were able, at some point during the vicious cycle of adversity, to focus on the meaningful purpose of some of their adversity. As such, they reconstruct their narrative into a narrative of meaningful endurance, thereby escaping the vicious cycle between health problems and unemployment. Just like Assila and Anna, all women whose life story could be characterized as a narrative of meaningful endurance were able to put a halt to the negative chain reaction they experience. Adversity, like health problems or unemployment, are still a part of life, however, it is easier to *endure* hardship if narrated to serve a meaningful purpose. In conclusion, the life stories of our respondents support that narratives of meaningful endurance enable women to escape a vicious cycle between health problems and unemployment by halting the negative chain reaction of adversity through meaningful reconstruction of adversity.

### Narratives in transition

These two types of narratives are so-called ideal-types placed on a continuum. Narratives that were more difficult to categorize show how women are actively engaged in reconstructing and reshaping their life stories. These narratives seem to be *narratives in transition*. Women narrating these stories reflect on how they recently started to grasp and make sense of how certain events in their life are related. A process of (meaningful) reconstruction of the life story is set in motion. By retelling the past, these women develop new perspectives on the present and the future.

Lisa, a 39-year-old Dutch woman, for example tells about how she started volunteering at a community center after suffering from a burnout. *“In the beginning, I was really afraid to make mistakes, I didn’t even dare to pick up the phone, pfff, I thought I am so not going to do that, even though I was doing that for over 15 years before in my previous job and I used to be great at that type of work.* […] *Yes, actually, not very long ago huh? You know, well, if I look back I really think I have grown a lot in the last months. I am not so insecure anymore. And also, you know, all these women that come here and are going through like this debt restructuring program or have psychological issues. I see where they are, where I was before, and now I can help them a little, and also see how far I got, do something valuable instead of sitting at home. When you say like, it was only 6 months ago, then I think by myself, wow! I don’t reflect on that enough. I have seen the lowest point, now I’m on my way back up. Maybe it was not for nothing, all of this, maybe, I don’t know”.* In the previous part of this interview Lisa is mostly telling about her burnout and her debt issues. While telling her life story she reflects on the progress she made in this period, it creates a shift in the way she narrates. Instead of focusing on her own hardship and suffering, she starts focusing on the valuable work she is doing and the progress she made, and hesitantly - “maybe, I don’t know”- starts the meaningful reconstruction of her past experiences. This excerpt highlights how meaningful reconstruction is an on-going process and how meaning can change over time.

### The relation between SOC and narratives of meaningful endurance

We have argued that narratives are a potential mechanism through which SOC contributes to escaping the vicious cycle between health problems and unemployment. The life story interviews highlight how, through narratives of meaningful endurance, the vicious cycle between health problems, unemployment and other negative developments can be broken, through the meaningful reconstruction of previous hardship. Hence, we would expect that individuals with a strong SOC are more inclined to tell narratives of meaningful endurance. The analysis of covariance shows that there is a significant difference in level of SOC across individuals with different types of narratives after controlling for age and ethnic background (F (2,47) = 20.228, *p* < .001). The covariate, age, is not significantly related to level of SOC, (F (1,47) = .763, *p* = .387). The covariates for ethnic background were also not significantly related to SOC, being from Surinamese (F (1,47) = .031, *p* = .861), Turkish (F (1,47) = .355, *p* = .554) or Moroccan (F (1,47) = .058, *p* = .811) background was not significantly related to the level of SOC (with Dutch women as a reference group). Planned contrasts show that individuals with a narrative of meaningful endurance have a significantly higher level of SOC than individuals with narratives of non-directional distress (+ 22.285, *p* < .001, 95% CI: 15.19–29.38), and than individuals with narratives in transition (+ 12.492, *p* = .015, 95% CI: 2.499–22.49). However, there is no significant difference between individuals with a narrative of non-directional distress and individuals with a narrative in transition (9.792, *p* = .066, 95% CI: −.67–20.26). It appears that indeed individuals with a stronger SOC are more likely to narrate their life stories in a narrative of meaningful endurance.

### The relation between SOC’s components and aspects of narrative

The life history interviews show that not only are individuals with a stronger SOC more likely to narrate their life stories in a narrative of meaningful endurance, the different components of SOC appear to be related to the subsequent characteristics of this type of narrative. It appears that the three components of SOC, comprehensibility, manageability and meaningfulness, play a role in enabling individuals to achieve structure, authorship and meaningful reconstruction in their life narratives (see Table 1).

**Table 1:** Overview characteristics and differences between the Sense of Coherence, narratives of meaningful endurance and narratives of non-directional distress

SOC components	Narrative characteristics		
		Narratives of meaningful endurance	Narratives of non-directional distress
Comprehensibility	Structure	Structured	Fragmented
	Causality	Focus on causal relation between events	No imposed causal order, random occurrence
Manageability	Authorship & agency	Self is presented as the main author of life story, focus on agency	Self is presented as not having authorship over the life-story, focus on lack of agency
Meaningfulness	Narrating adversity	Adversity is reinterpreted as meaningful, contributing to some purpose	Adversity is narrated as disruptive and serving no purpose

First, the definition of comprehensibility is “the extent to which one perceives the stimuli that confront one, deriving from the internal and external environments, as making cognitive sense, as information that is ordered, consistent, structured, and clear, rather than as noise – chaotic, disordered, random, accidental, inexplicable” (Antonovsky, 1987, p. 6). Hence, comprehensibility, perceiving life as ordered and making cognitive sense, enables individuals in applying structure and causal order in their life narratives. Second, manageability is conceptualized as feeling able to deal with arising issues and having the resources to do so (Antonovsky, 1987). We argue that individuals who score high on manageability are not only able to manage arising issues, but also feel able to manage how they perceive and present their life story. They perceive and present themselves as a shaping force of their life narratives, or in other words, as having authorship. Third, Antonovsky describes the meaningfulness component as “the extent to which one feels that life makes sense emotionally, that at least some of the problems and demands posed by living are worth investing energy in, are worthy of commitment and engagement”. Taking a life course perspective, individuals with strong meaningfulness will not only perceive arising issues as worthy of commitment and engagement, previously experienced adversity is perceived as worthy and valuable to have lived through, resulting in a conception of one’s life story which makes sense emotionally. In other words, the meaningfulness component of SOC enables individuals to perceive and reconstruct the past, present and future in a meaningful way. In conclusion, narratives of meaningful endurance “reconstruct the past and anticipate the future in such a way as to provide life with identity, meaning, and coherence” (McAdams, 2006, p. 110). The process of narrating one’s life story as a narrative of meaningful endurance emerges as a mechanism through which SOC, by reinterpreting life events, aids the escape of vicious cycles between health problems and unemployment.

All individuals are, at some point during their life course, confronted with adversity, and for all individuals this will complicate sustaining a narrative of meaningful endurance. For individuals with a strong SOC, due to their strong comprehensibility, manageability and meaningfulness, developing structure, authorship and meaningful reconstruction in their life narratives is more easily achieved than for individuals with a weak SOC. For individuals with a weak SOC, with a lack of comprehensibility, manageability and meaningfulness, developing a narrative of meaningful endurance is like fighting an uphill battle. SOC seems to operate through the coping process as Antonovsky predicted, and narratives are the more specific method of coping through which SOC operates.

## Discussion

The purpose of this study was to examine how women escape the vicious cycle between health problems and unemployment by using the theory of the Sense of Coherence. Previous research has predominantly focused on how SOC influences different health related outcomes, but not so much on *how* SOC works or *whether* SOC is applicable outside the domain of health. Hence, we also aimed to increase our understanding of the mechanisms through which SOC operates and its applicability outside the domain of health research. Our findings show that women can escape the vicious cycle between health problems and unemployment through the meaningful reconstruction of adversity. Women can put a halt on the on-going negative chain reaction through focusing on the meaning and purpose of adversity. We named such life-stories narratives of meaningful endurance. In addition, we found that that the three respective components of SOC - comprehensibility, manageability and meaningfulness - enable the attainment of structure, authorship and meaningful reconstruction, the three defining characteristics of a narrative of meaningful endurance. Moreover, we found that a higher level of SOC is associated with narratives which can be characterized as a narrative of meaningful endurance.

Our findings show that the narratives of the women in this study vary with respect to structure, authorship and meaningful reconstruction, according to which they can be placed on a continuum between what we call *narratives of meaningful endurance* and *narratives of non-directional distress*. The different narrative types we found in this study show a resemblance with previous findings about different illness narratives. Narratives of non-directional distress seem to correspond closely to Frank’s (1998) *chaos stories*, both are unstructured, chaotic and focus on perpetual suffering. Narratives of meaningful endurance, on the other hand, seem to reflect what Frank names the *quest story*, with its focus on the lessons that can be learned from illness. However, there are also striking differences. Frank mostly focuses on how illness threatens identity, with a change or restitution of an individual’s identity as a defining characteristic of the narratives. However, in this study we did not focus on the transformation or restoration of identity, instead we focused on the meaningful reconstruction of hardship in order to stop the negative chain reaction of adversity. In some cases, this can be related to identity, as finding meaning and purpose in life experiences is related to how we perceive and construct the self. Yet, the meaningful reconstruction of hardship does not necessarily result in the restoration or transformation of identity. The narrative of Anna, for example, shows how her struggles about her identity were actually the cause of developing psychological health problems, instead of the other way around. In addition, in Frank’s work the focus is on the narration of illness, our focus was on general life stories in which employment or adversary life events were sometimes of much greater importance than illness.

The process of meaningful reconstruction which we described using our own terms, could also be described as *biographical work to achieve biographical reinforcement* (Carricaburu & Pierret, 1995), *legitimation* (Bury, 1991), *knitting together ruptured identities (surturing)* (Riessman, 2015), and *narrative reconstruction* (Williams, 1984). These terms describe similar processes of reconstructing the life story through narration with the aim to achieve a meaningful and coherent story. The proliferation of terms to describe these similar endeavors reflect the rich diversity in how individuals achieve this aim. When focusing mostly on the restoration or transformation of identity, as these studies do, biographical work and surturing ruptured identities are more suitable terms. In our case, with the main focus on escaping the vicious cycle between health problems and unemployment, irrespective of what happens to identity, we prefer meaningful reconstruction as a more suitable term. For further theoretical development, it is important to note these commonalities but to simultaneously appreciate the richness and variety in the processes described.

The second aim of this study was to expand the use of SOC outside the domain of health and to examine the mechanisms through which SOC operates. Our findings highlight the potential of using SOC theory outside the domain of health research. SOC, as a general orientation to life, does not only protect individuals’ health from risk factors. SOC also enables individuals to meaningfully reconstruct previous hardship and deal with arising issues so as to limit their negative impact on, for example, employment. SOC theory, with its encompassing character and focus on success factors, is a promising theory to apply on new issues and fields of study in future research. Secondly, we looked into whether SOC might operate through enabling individuals to narrate their life story in such a way that it prevents the negative reinforcing relation between health problems and unemployment to continue. Our findings (see Table 1) show that there is a striking resemblance between the three components of SOC and the three major characteristics of the types of narratives that we uncovered. The three components of SOC, comprehensibility, manageability and meaningfulness, enable individuals to attain structure, authorship and meaningful reconstruction in their life narratives. From this we infer that narratives of meaningful endurance are, as such, a mechanism through which SOC enables individuals to escape the vicious cycle between health problems and unemployment.

It could also be argued that the types of narratives we found are not a mechanism through which SOC operates, but a direct reflection of SOC, a way to ‘measure’ SOC in qualitative studies. Yet, having a narrative of meaningful endurance is not the same as having a strong SOC. The Sense of Coherence is a general orientation to life which stabilizes in early adulthood (Antonovsky, 1987). Narratives, on the other hand, refer to how individuals narrate their past, present and imagined future at a certain point in time, which may change over time. Narratives change over time, and any individual, with a strong or a weak SOC, will have difficulties maintaining a narrative of meaningful endurance when facing adversity. It is those individuals with a strong SOC who are more resistant to being pushed towards a narrative of non-directional distress and are better able to maintain a narrative of meaningful endurance when facing adversity. A strong SOC *enables* individuals to narrate their life story in a certain way which makes strong SOC individuals more likely to have a narrative of meaningful endurance. To illustrate, individuals who score high on the meaningfulness component of SOC perceive that life has a purpose and is meaningful. However, when a relative passes away, they lose their job or suffer from severe health problems, they may not perceive this adversity as serving some meaningful purpose. If you generally perceive life as meaningful, you are better able to narrate adversity as serving a meaningful purpose, but this is not a given.

A potential limitation of this study may be the diverse nature of the sample with respect to ethnic background. However, despite the ethnic diversity in the sample, we were able to place all narratives of the women in our sample on the continuum between these two types of narratives. Therefore, we believe that the diverse nature of the sample strengthens our findings. Another potential limitation of this study is that some women were not native Dutch speakers. We aimed to limit the effect of language difficulties on the results by offering the use of an interpreter. Three respondents decided to make use of an interpreter during the interview and the subsequent filling in of the Orientation to Life Questionnaire. However, non-native speakers who did not make use of an interpreter did not differ in how they narrated their life stories. Despite the obstacles non-native speakers experienced in speaking Dutch, they managed, through body language, tone, occasionally using translation tools and meaningful silences to narrate their life story in all its richness. Here it is also important to note that the ways in which women created structure, causal order, authorship and applied meaningful reconstruction varied greatly. However, irrespective of *how* women achieved these elements of their life narratives, the narratives could all be placed on the continuum between narratives of meaningful endurance and narratives of non-directional distress.

In this study we explore the mechanisms through which SOC operates and the use of SOC outside the realm of health. We find support for two ‘grand’ narratives through which SOC operates: *narratives of non-directional distress* and *narratives of meaningful endurance*. These findings may also be of interest for interventions directed at promoting health and/or employment. The results of several previous studies show that various types of interventions, of varying length and among different populations, are able to increase the level of SOC (Delbar & Benor, 2001; Forsberg, Björkman, Sandman, & Sandlund, 2010; Vastamäki, Moser, & Paul, 2009; Weissbecker et al., 2002; Ying, 1999). In relation to ethnic minority women, a recent study found that the processes of migration and integration may be a potential threat to developing a strong Sense of Coherence among Turkish and Moroccan women living in the Netherlands. Moreover, this study highlights how women's General Resistance Resources, such as social support, religion and collective stories, could promote developing a strong SOC (Slootjes, Keuzenkamp, & Saharso, 2017). Based on the results of these studies and our results, we recommend focusing on narrative techniques and strengthening SOC as promising ways to enable ethnic minority women to escape the vicious cycle between health problems and unemployment.

## Footnotes

1 For a more elaborate review of the literature on health and employment of ethnic minority women in the Netherlands see Slootjes (2017).

2 The empirical studies referred to in this review study, look into the effect of SOC as a unified concept on various health-related outcomes.

3 We use the definition of health of the WHO: “Health is a state of complete physical, mental and social well-being and not merely the absence of disease or infirmity” as stated in the Constitution of the World Health Organization from 1946.

4 In this study, we consider individuals without a paid job unemployed. We focus mostly on (un)employment, yet other forms of participation like volunteer work or being enrolled in education have a similar relation to health problems. However, these activities oftentimes have a more non-committal character. Therefore, health problems likely have a weaker effect on these forms of participation.

5 The SOC-13 survey has been used in at least 33 languages in over 32 countries and has been validated in over 300 empirical studies (Eriksson & Lindström, 2005). The translated Dutch version of the SOC-13 survey has been validated in a study by Jellesma et al. (2006).
